# Subclinical hypothyroidism and height loss according to free thyroxine levels: a prospective study

**DOI:** 10.1265/ehpm.25-00362

**Published:** 2025-12-15

**Authors:** Yuji Shimizu, Nagisa Sasaki, Yuko Noguchi, Mutsumi Matsuu-Matsuyama, Shin-Ya Kawashiri, Hirotomo Yamanashi, Kazuhiko Arima, Seiko Nakamichi, Yasuhiro Nagata, Takahiro Maeda, Naomi Hayashida

**Affiliations:** 1 Department of General Medicine, Nagasaki University Graduate School of Biomedical Sciences, Nagasaki 852-8501, Japan; 2 Epidemiology Section, Division of Public Health, Osaka Institute of Public Health, Osaka 537-0025, Japan; 3 Department of Community Medicine, Nagasaki University Graduate School of Biomedical Sciences, Nagasaki 852-8523, Japan; 4 Division of Strategic Collaborative Research, Atomic Bomb Disease Institute, Nagasaki University, Nagasaki, 852-8523, Japan; 5 Leading Medical Research Core Unit, Nagasaki University Graduate School of Biomedical Sciences, Nagasaki 852-8523, Japan; 6 Department of Public Health, Nagasaki University Graduate School of Biomedical Sciences, Nagasaki 852-8501, Japan; 7 Nagasaki University Health Center, Nagasaki 852-8521, Japan

**Keywords:** Free thyroxine, Height loss, Subclinical hypothyroidism, Thyroid hormone

## Abstract

**Background:**

Subclinical hypothyroidism (SCH) has been reported to be associated with lower endothelial progenitor (CD34-positive) cell count, whereas an inverse association between circulating CD34-positive cell count and height loss is documented. Reports indicate height loss to be associated with all-cause mortality, and a higher CD34-positive cell count has been shown to predict longer life. Therefore, evaluating the association between SCH and height loss provides mechanistic insights underlying the association between height loss and mortality risk.

**Methods:**

A prospective study involving 1,599 participants with normal free triiodothyronine (T3) and free thyroxine (T4) levels was conducted to determine the association between SCH and height loss.

Since the free T4 level influences the supply of active thyroid hormone (free T3), the analysis was stratified by the median free T4 level. Height loss was defined as the highest quintile of annual height decrease.

**Results:**

SCH was positively associated with height loss in participants with low-normal free T4 levels (below the median), but not in those with high-normal free T4 levels (at or above the median). After adjusting for sex, age, free T3 level, atherosclerosis, and known cardiovascular risk factors, the adjusted odds ratios (95% confidence interval) for height loss were 1.88 (1.02, 3.47) and 1.92 (1.02, 3.62) in the low-normal free T4 group. The corresponding values in the high-normal free T4 group were 0.37 (0.08, 1.69) and 0.43 (0.09, 1.97).

**Conclusion:**

SCH could influence height loss, and free T4 might influence the association between SCH and height loss in euthyroid individuals. These results clarify the mechanisms underlying the association between height loss and mortality risk.

## 1. Introduction

Hypertension is a well-known cardiovascular risk factor affecting vascular health [1]. Endothelial function and the vascular network must be maintained to ensure vascular health. Hypertension is positively associated with height loss, as defined by being in the highest quintile of annual height decrease among Japanese workers aged 40 to 74 years [2].

A close relationship has been reported between atherosclerosis and hypertension [3]. Atherosclerosis has also been demonstrated to be positively associated with height loss, as defined by being in the highest quintile of height decrease per year among Japanese individuals aged 60 to 89 years who undergo annual health checkups [4].

Higher glycated hemoglobin (HbA1c) levels within the normal range have been reported as independent risk factor for hypertension [5], whereas height loss, defined as being in the highest quintile of height decrease per year, has also been shown to be positively associated with normal HbA1c [6]. In addition, a slightly high HbA1c level increases all-cause and cardiovascular disease mortality among Japanese individuals [7], whereas height loss starting in middle age predicts increased mortality in the elderly [8].

Therefore, the vascular health status may play a crucial role in height loss in the general population. Additionally, our previous study on Japanese men aged 60 to 69 years revealed a significant inverse association between circulating CD34-positive cell (endothelial progenitor cell) count, which could indicate endothelial repair activity [9, 10], and height loss, defined as being in the highest quartile of height decrease per year [11]. Increased endothelial repair activity, which could have a beneficial influence on vascular health, may prevent height loss.

Since subclinical hypothyroidism (SCH) decreases the number of endothelial progenitor cells, while thyroxine (T4) treatment increases the number of circulating endothelial progenitor cells [12], SCH may be associated with height loss, and serum free T4 levels might influence the association between SCH and height loss in the general population with normal thyroid function (i.e., individuals with free triiodothyronine [T3] and free T4 levels within the normal range).

Height loss has been reported to be associated with all-cause mortality [13], and a higher CD34-positive cell count predicts longer life [14]. Therefore, evaluating the association between SCH and height loss helps clarify the mechanisms underlying the association between height loss and mortality risk.

This study aimed to elucidate the association between SCH and height loss in relation to free T4 levels, and therefore, a prospective study in a general euthyroid population was conducted.

## 2. Materials and methods

### Study population

The methods related to risk surveys, including thyroid examinations, used in this study have been described elsewhere [15–17].

This study was approved by the ethics committee of Nagasaki University Graduate School of Biomedical Sciences (project registration number 14051404). Written informed consent was obtained from all the participants. Consent forms were used to ensure that the participants understood the objectives of the study while obtaining informed consent. All procedures involving human participants were performed in accordance with the ethical standards of the Institutional Research Committee and the Declaration of Helsinki (2024) for comparable ethical standards.

This prospective study included 1,883 Japanese individuals aged 40–74 years from the town of Saza in western Japan. These individuals underwent annual medical check-ups in 2014, as recommended by the Japanese government.

To avoid the influence of thyroid disorders, individuals were excluded if they had a history of thyroid disease (n = 60); had missing data on thyroid function such as thyroid stimulating hormone (TSH), free T3, and free T4 levels (n = 17); or had levels outside of the normal range for T3 (2.1–4.1 pg/mL) or T4 (1.0–1.7 ng/dL) (n = 77).

Individuals without data on body mass index (BMI) (n = 1) or atherosclerosis status (n = 1) were additionally excluded. Individuals were also excluded if data on drinking (n = 1) or smoking status (n = 2) were missing. To calculate the height decrease per year for the present study, at least two height measurements (baseline and endpoint) were necessary during the observation period. Therefore, participants without height measured during 2015–2022 (endpoint) were excluded from the analysis (n = 125).

The analysis included 1,599 participants with a mean age of 60.8 years (standard deviation (SD): 8.9; range: 40–74 years). Mean follow-up was 5.2 years (SD, 2.3 years; interquartile range, 3.0–7.1 years).

### Data collection and laboratory measurements

Trained interviewers obtained information on the clinical characteristics. An automatic body composition analyzer (BF-220; Tanita, Tokyo, Japan) was used to calculate BMI (kg/m^2^) after measuring height and weight. A participant was considered to have height loss if he or she was in the highest quintile of height decrease per year, as described in our previous studies [2, 6, 18].

Blood pressure (systolic and diastolic) was measured in the sitting position using a blood pressure measuring device (HEM-907; Omron, Kyoto, Japan) after at least 5 minutes of rest. High blood pressure was defined as systolic blood pressure ≥140 mmHg, diastolic blood pressure ≥90 mmHg, and/or anti-hypertensive medication use.

Fasting blood samples were collected from all participants. TSH, free T3, and free T4 levels were measured using chemiluminescent immunoassays (LSI Medience Corporation, Tokyo, Japan). The normal ranges for free T3 (2.1–4.1 pg/mL), free T4 (1.0–1.7 ng/dL), and TSH (0.39–4.01 µIU/mL) based on this method are described elsewhere [19]. HbA1c and high-density lipoprotein cholesterol (HDLc) levels were measured by SRL Inc. (Tokyo, Japan) using standard procedures. Individuals with a serum TSH concentration >4.01 µIU/mL were defined as having SCH.

Experienced vascular technicians measured the carotid intima-media thickness (CIMT) using a LOGIQ Book XP device with a 10-MHz transducer (GE Healthcare, Milwaukee, WI, USA). The maximum CIMT values for the left and right common carotid arteries were calculated using a semi-automated digital edge-detection software (Intimascope; Media Cross, Tokyo, Japan) as described previously [20, 21].

The maximum right and left CIMT values, which did not include plaque measurements, were calculated. The maximum CIMT value was used for the analysis. Since a previous study reported CIMT <1.1 mm as normal, we defined atherosclerosis as CIMT ≥1.1 mm [1, 9, 22].

### Statistical analysis

The clinical characteristics of the study participants were compared according to SCH status while accounting for free T4 levels. Data are presented as means ± SD or n (%), except for TSH. Since TSH values had a skewed distribution, the median (interquartile range) is presented. Trend tests were conducted.

Logistic regression models were used to calculate odds ratios (ORs) and 95% confidence intervals (CIs) to determine the associations between free T4 levels and height loss, and between SCH and height loss.

The following four methods were used to adjust for confounding factors: Model 1 adjusted for sex and age. Model 2 adjusted for sex, age, and free T3 levels because free T3 is the active form of the thyroid hormone. Model 3 adjusted for sex, age, free T3 levels, and atherosclerosis. Aggressive endothelial repair, which leads to atherosclerosis, requires circulating CD34-positive cells [9, 10, 23], which are associated with height loss [11]. Therefore, atherosclerosis can cause height loss [4]. Model 4 adjusted for sex, age, and known cardiovascular risk factors that may have influenced height loss. These included hypertension, high BMI (25 kg/m^2^≤), slightly higher HbA1c (5.3–5.5%), higher HbA1c (5.6–6.4%), high HbA1c (6.5%≤), daily drinking, current smoking, and HDLc level. High BMI (25 kg/m^2^≤) is positively associated with height loss [18, 24]. Hypertension is an independent risk factor of height loss [2]. Circulating CD34-positive cells have been shown to influence the status of hypertension and atherosclerosis [3]. HbA1c levels within the normal range are reportedly associated with height loss. Compared with HbA1c <5.6%, HbA1C (5.3–5.5%), HbA1c (5.6–6.4%), and high HbA1c (≤6.5%) are reported to be positively associated with height loss among Japanese individuals [6]. Although hemoglobin is inversely associated with height loss [18], we did not have data on hemoglobin levels in this study. Since smoking and drinking status could influence hemoglobin levels [25, 26], these could act as confounders in the present study. Current smoking was previously found to be an independent risk factor for height loss in Japanese men [27]. In Japanese individuals, eating speed, which is associated with height loss and high BMI [24], has been reported to be associated with low HDLc levels (<40 mg/dL in men and <50 mg/dL in women) [28].

In general, to compensate for reduced thyroid hormone production, the need for TSH is increased. Therefore, to maintain thyroid hormone levels within the normal range in SCH, serum concentrations of TSH should be increased. However, even if thyroid hormone levels are within the normal range, individuals with low-normal free T4 levels (below the median) might have a more severe thyroid hormone deficit than individuals with high-normal free T4 levels (at or above the median) because T3 production by the thyroid gland is limited, whereas T4 is produced almost daily by the thyroid gland [29]. Therefore, to evaluate the association between SCH and height loss, analyses stratified by the median free T4 levels were conducted in the present study.

For the sensitivity analysis, we performed the main analysis separately according to sex. We also analyzed the association between SCH and height loss, defined as the highest sextile height decrease per year.

All statistical analyses were performed using SAS for Windows (version 9.4; SAS Inc., Cary, NC, USA). Statistical significance was set at p < 0.05.

## 3. Results

### Clinical characteristics of the study population according to T4 level and SCH

Table 1 describes the clinical characteristics of the study population according to free T4 levels. People with high-normal T4 levels had significantly higher free T3 levels, were significantly more likely to be men, have hypertension, have low HDL levels, be daily drinkers, and have significantly lower TSH levels than those with low-normal free T4 levels.

**Table 1:** Characteristics of the study population by levels of free T4

	**Free T4**		**p**

	**Low ** **(<median)**	**High ** **(median≤)**	
No. of participants	857	742	
Men, %	30	44.3	<0.001
Age, year	60.9 ± 9.0	60.7 ± 8.8	0.542
TSH, (0.39–4.01) µIU/mL	1.69 [1.16, 2.47]*^1^	1.47 [1.02, 2.09]*^1^	<0.001*^2^
free T3, (2.1–4.1) pg/mL	3.1 ± 0.3	3.3 ± 0.3	<0.001
free T4, (1.0–1.7) ng/dL	1.13 ± 0.07	1.39 ± 0.108	<0.001
Hypertension, %	36.9	43.4	0.008
High BMI (25 kg/m^2^≤), %	21.7	24.9	0.127
Slightly higher (5.3–5.5%), %	33.6	34.0	0.881
Higher HbA1c (5.6–6.4%), %	37.5	34.2	0.181
High HbA1c (6.5%≤), %	6.7	7.4	0.552
Low HDLc (<40 mg/dL), %	4.2	8.0	0.002
Daily drinker, %	35.6	43.9	<0.001
Current smoker, %	11.7	14.3	0.119
Atherosclerosis, %	9.9	9.4	0.744

TSH; thyroid-stimulating hormone, T3; triiodothyronine, T4: thyroxine, BMI: body mass index. Values are mean ± standard deviation or n (%). *1: Values are median [the first quartile, third quartile]. *2: Logarithmic transformation was used. Normal range of measurements are ( ). Median values of free T4 were 1.3 ng/mL for men and 1.2 ng/mL for women.

### Clinical characteristics of the study population according to T4 level and SCH

Clinical characteristics of the study population according to free T4 levels and SCH status are shown in Table 2. Among participants with low-normal free T4 levels, those with SCH had a significantly higher prevalence of hypertension and high HbA1c and significantly lower serum free T4 concentrations than those without SCH. Among individuals with high-normal free T4 levels, those with SCH had a significantly higher prevalence of low HDLc levels and atherosclerosis than those without SCH.

**Table 2:** Characteristics of the study population by T4 level and SCH

	**Free T4**					

	**Low (<median)**			**High (median≤)**		

	**SCH**		**p**	**SCH**		**p**
	
	**(−)**	**(+)**		**(−)**	**(+)**	
No. of participants	791	66		720	22	
Men, %	29.5	36.4	0.240	44.2	50.0	0.588
Age, year	60.8 ± 9.0	62.9 ± 8.7	0.059	60.7 ± 8.8	61.0 ± 9.3	0.856
TSH, (0.39–4.01), µIU/mL	1.60 [1.14, 2.25]*^1^	5.31 [4.60, 6.10]*^1^	<0.001*^2^	1.44 [1.01, 2.01]*^1^	4.71 [4.25, 6.24]*^1^	<0.001*^2^
free T3, (2.1–4.1), pg/mL	3.1 ± 0.3	3.1 ± 0.3	0.182	3.3 ± 0.3	3.3 ± 0.3	0.646
free T4, (1.0–1.7), ng/dL	1.13 ± 0.07	1.10 ± 0.09	0.004	1.39 ± 0.11	1.40 ± 0.10	0.659
Hypertension, %	35.8	50.0	0.021	42.9	59.1	0.132
High BMI (25 kg/m^2^≤), %	21.5	24.2	0.603	24.4	40.1	0.079
Slightly higher (5.3–5.5%), %	34.6	21.2	0.027	34.0	31.8	0.830
Higher HbA1c (5.6–6.4%), %	36.7	47.0	0.097	34.0	40.9	0.504
High HbA1c (6.5%≤), %	5.6	19.7	<0.001	7.1	18.2	0.050
Low HDLc (<40 mg/dL), %	4.0	6.1	0.434	7.5	22.7	0.009
Daily drinker, %	36.0	30.3	0.351	44.4	27.3	0.110
Current smoker, %	12.1	6.1	0.140	14.4	9.1	0.480
Atherosclerosis, %	9.6	13.6	0.293	9.0	22.7	0.030

SCH; subclinical hypothyroidism, TSH; thyroid-stimulating hormone, T3; triiodothyronine, T4: thyroxine, BMI: body mass index. Values are mean ± standard deviation or n (%). *1: Values are median [the first quartile, third quartile]. *2: Logarithmic transformation was used. The normal range of measurements is ( ). Median values of free T4 were 1.3 ng/mL for men and 1.2 ng/mL for women.

### Association between T4 levels and height loss

No significant association was found between free T4 levels and height loss in Models 1–4 (Table 3).

**Table 3:** Association between free T4 and height loss

	**1 SD increment of free T4**	**p**
No. of participants	1,599	
No. of cases (%)	319	
Model 1	0.97 (0.85, 1.10)	0.617
Model 2	1.01 (0.88, 1.15)	0.928
Model 3	1.01 (0.88, 1.15)	0.935
Model 4	0.97 (0.85, 1.10)	0.627

T4; thyroxine, SD; standard deviation. Model 1: Adjusted only for sex and age. Model 2: Adjusted for sex, age, and free triiodothyronine (T3). Model 3: Factors adjusted for Model 2 plus atherosclerosis. Model 4: Adjusted for sex, age, hypertension, high body mass index, slightly higher HbA1c, higher HbA1c, high HbA1c, daily drinking, current smoking, and low HDL-cholesterol. 1SDs of free thyroxine (T4) were 0.15 ng/mL for men and 0.17 ng/mL for women.

### Association between subclinical hypothyroidism (SCH) and height loss among all participants

Table 4 shows the association between SCH and height loss in all participants. No significant associations were observed in Models 1–4.

**Table 4:** Association between SCH and height loss

	**SCH**		**p**

	**(−)**	**(+)**	
No. of participants	1,511	88	
No. of height loss (%)	299 (19.8)	20 (22.7)	
Model 1	Ref	1.40 (0.81, 2.42)	0.226
Model 2	Ref	1.36 (0.78, 2.35)	0.275
Model 3	Ref	1.37 (0.79, 2.38)	0.258
Model 4	Ref	1.43 (0.82, 2.50)	0.207

SCH; subclinical hypothyroidism, Ref: reference. Model 1: Adjusted only for sex and age. Model 2: Adjusted for sex, age, and free triiodothyronine (T3). Model 3: Factors adjusted for Model 2 plus atherosclerosis. Model 4: Adjusted for sex, age, hypertension, high body mass index, slightly higher HbA1c, higher HbA1c, high HbA1c, daily drinking, current smoking, and low HDL-cholesterol.

### Association between subclinical hypothyroidism (SCH) and height loss while accounting for free thyroxine (T4) levels

The associations between SCH, height loss, and free T4 levels are shown in Table 5. Among participants with low-normal free T4 levels, SCH was significantly associated with height loss. The sex- and age-adjusted OR (95% CI) for height loss was 1.90 (1.32, 3.50). This association persisted even after further adjustments for free T3 levels (Model 2), free T3 levels, atherosclerosis (Model 3), and other known confounding factors (Model 4). Among the participants with high-normal free T4 levels, SCH was inversely associated with height loss in Models 1–4, although the results were not statistically significant.

**Table 5:** Association between SCH and height loss by free T4 levels

	**Free T4**					

	**Low (<median)**			**High (median≤)**		

	**SCH**		**p**	**SCH**		**p**
	
	**(−)**	**(+)**		**(−)**	**(+)**	
No. of participants	791	66		720	22	
No. of height loss (%)	156 (19.7)	18 (27.3)		143 (19.9)	2 (9.1)	
Model 1	Ref	1.90 (1.32, 3.50)	0.039	Ref	0.38 (0.08, 1.71)	0.207
Model 2	Ref	1.87 (1.01, 3.44)	0.045	Ref	0.36 (0.08, 1.67)	0.194
Model 3	Ref	1.88 (1.02, 3.47)	0.043	Ref	0.37 (0.08, 1.69)	0.198
Model 4	Ref	1.92 (1.02, 3.62)	0.044	Ref	0.43 (0.09, 1.97)	0.277

SCH; subclinical hypothyroidism, Ref; reference, T4; thyroxine. Model 1: Adjusted only for sex and age. Model 2: Adjusted for sex, age, and free triiodothyronine (T3). Model 3: Factors adjusted for Model 2 plus atherosclerosis. Model 4: Adjusted for sex, age, hypertension, high body mass index, slightly higher HbA1c, higher HbA1c, high HbA1c, daily drinking, current smoking, and low HDL-cholesterol. Median values of free T4 were 1.3 ng/mL for men and 1.2 ng/mL for women.

### Sensitivity analysis

For the sensitivity analysis, we performed sex-specific analyses of the free T4 level-specific association between SCH and height loss. We found similar associations. Among participants with low-normal free T4 levels, the age-adjusted OR (95% CI) for height loss of SCH compared with SCH was 1.39 (0.50, 3.87) for men (n = 257) and 2.30 (1.08, 4.90) for women (n = 600). Among participants with high-normal free T4 levels, the corresponding values were 0.37 (0.05, 3.06) for men (n = 329) and 0.40 (0.05, 3.47) for women (n = 413).

To assess sensitivity, we performed the main analysis again with height loss defined as being in the highest sextile of the annual height decrease. The same results were obtained. Among participants with low-normal free T4 levels, the age-adjusted OR (95% CI) for height loss of SCH compared with no SCH was 1.97 (1.03, 3.78) for Model 1, 1.91 (1.00, 3.67) for Model 2, 1.92 (1.00, 3.69) for Model 3, and 2.02 (1.03, 3.96) for Model 4. Among participants with high-normal free T4 levels, the corresponding values were 0.21 (0.03, 1.63), 0.20 (0.03, 1.59), 0.20 (0.03, 1.58), and 0.25 (0.03, 1.92).

## 4. Discussion

The major finding of the present study was that SCH was significantly and positively associated with height loss in participants with low-normal levels of free T4 but not in those with high-normal levels of free T4.

A previous prospective study of 363 Japanese men aged 60–69 years over two years found that, independent of known cardiovascular risk factors, circulating endothelial progenitor (CD34-positive) cell count was significantly inversely associated with height loss, which was defined as being in the highest quartile of height decrease per year [11]. Since circulating CD34-positive cell counts indicate endothelial maintenance activity, a higher circulating CD34-positive cell count may have a beneficial effect in maintaining vascular health [9, 10]. Circulating CD34-positive cell counts were reported to be inversely associated with cardiovascular disease-related mortality [30, 31], while height loss was reported to be positively associated with cardiovascular disease [32, 33].

In addition, a case-control study of 20 patients with SCH and healthy controls reported lower endothelial progenitor cell counts in participants with SCH than in healthy controls [12]. This study also reported that T4 treatment increased the endothelial progenitor cell count.

These studies indicate that SCH could be a risk factor for height loss in the general population and that free T4 levels influence the association between SCH and height loss. In the present study, a significant association between SCH and height loss was observed only in participants with low-normal free T4 levels.

Daily production of the active type of thyroid hormone (T3) mostly originates from the peripheral deiodination of T4 [29]. Therefore, the ratio of free T3 to free T4 (free T3/free T4), which is a marker of peripheral thyroxine deiodination, indicates thyroid hormone activity [34].

In this study, participants with high-normal free T4 levels had significantly lower TSH values and significantly higher free T3 levels than those with low-normal free T4 levels (Table 1). These results indicate that individuals with low-normal free T4 levels had comparatively lower thyroid hormone activity but higher thyroid hormone demand than those with high-normal free T4 levels.

Although TSH levels were significantly higher in participants with low free T4 levels than in those with high free T4 levels (Table 1), no significant association was found between free T4 levels and height loss (Table 3). Therefore, free T4 levels may not predict the risk of height loss.

As shown in Table 2, in the present study, among participants with low-normal free T4 levels, serum levels of free T4 were slightly but significantly lower in those with SCH than in those without SCH. Among those with high-normal free T4 levels, no significant differences were observed in the prevalence of SCH. Therefore, among participants with low-normal free T4 levels, the presence of SCH might indicate a strong activation of peripheral deiodination, which reduces the concentration of free T4 in the peripheral blood. Among participants with low free T4 levels, the presence of SCH could indicate a comparatively higher activity of peripheral thyroid hormones stimulated by vascular injury.

In the present study, which was statistically underpowered, SCH was associated with a lower OR for height loss among participants with normal free T4 levels.

In these participants, the thyroid gland itself may have sufficient capacity to produce thyroid hormones, and the presence of SCH stimulates the production of thyroid hormones, which may have a beneficial effect in preventing height loss.

The evaluation of atherosclerosis using CIMT indicates that the results of aggressive endothelial repair are reflected in CIMT, as aggressive endothelial repair increases CIMT [35]. Hypertension stimulates endothelial repair [9]. In this study, among participants with low free T4 levels, the prevalence of hypertension was significantly higher among those with SCH than among those without SCH, and the prevalence of atherosclerosis was higher but not significantly (Table 2). Among the participants with high free T4 levels, the prevalence of hypertension was higher, but not significantly, among those with SCH than among those without SCH. The prevalence of atherosclerosis was significantly higher in the patients with SCH than in those without SCH (Table 2). Progression of CIMT partly indicates the prevention of hypertension [1]. Therefore, these associations partly indicate that for low and high levels of free T4, patients with SCH require endothelial repair. These associations also indicate that insufficient endothelial repair influences individuals with SCH and low free T4 levels more strongly than those with SCH and high free T4 levels.

### Strength and limitations

One clinical implication of the present study is that even among participants with normal thyroid hormone levels, free T4 levels may affect the association between SCH and height loss. Although further investigation with a larger sample size is required, the present findings suggest that SCH could have a beneficial effect on vascular health maintenance if serum-free T4 levels are in the high-normal range. Furthermore, this study indicated that even when serum concentrations of thyroid hormones are within the normal range, thyroid function can influence height loss in the general population. The present findings also help clarify the mechanisms underlying vascular health and height loss in the general population.

The limitations of this study warrant further consideration. As in our previous studies [4, 6, 11, 18, 24], an efficient cut-off point to define height loss has not been established. In this study, height loss was defined as the highest quintile of height decrease per year. However, our sensitivity analysis, based on the sextiles of height decrease per year, showed essentially the same association.

Owing to the small number of participants with SCH who had height loss and high normal free T4 levels (n = 2), the study was underpowered for analysis among participants with high-normal free T4 levels. However, a completely different association between SCH and height loss was observed in individuals with low versus those with high normal free T4 levels. A significant positive association was observed in those with low normal free T4 levels, and an insignificant but inverse association was observed in those with normal free T4 levels. The sample size in the present study prevented us from statistically evaluating meaningful sex-specific associations between SCH and height loss. However, sensitivity analysis showed essentially the same associations in men and women.

## 5. Conclusion

Among participants with low-normal free T4 levels, SCH was an independent risk factor for height loss in the general population. Among participants with high-normal free T4 levels, SCH prevented height loss; however, further investigation with a larger sample size is necessary. These findings provide new information that could help clarify the mechanisms underlying height loss in the general population.

## Abbreviations

SCH: subclinical hypothyroidism
T3: triiodothyronine
T4: thyroxine
HbA1c: glycated hemoglobin
TSH: thyroid-stimulating hormone
BMI: body mass index
SD: standard deviation
HDLc: high-density lipoprotein cholesterol
CIMT: carotid intima-media thickness
ORs: odds ratio
CIs: confidence interval

## Declarations

### Availability of data and material

For the studies described in this review, we cannot publicly provide individual data because of participant confidentiality considerations according to the ethical guidelines in Japan. Additionally, obtaining informed consent does not include a provision for publicly sharing the data. Qualified researchers have access to minimal datasets. Please contact the Office of Data Management at ritouken@vc.fctv-net.jp. Information on the data request is available at http://www.med.nagasaki-u.ac.jp/cm/.
